# Computed tomography differentiation of compact and cancellous bone tissue in short and sesamoid bones

**DOI:** 10.2478/raon-2025-0022

**Published:** 2025-04-11

**Authors:** Ziva Miriam Gersak, Irena Zupanic-Pajnic, Eva Podovsovnik, Vladka Salapura

**Affiliations:** Institute of Radiology, University Medical Centre Ljubljana, Ljubljana, Slovenia; Institute of Forensic Medicine, Faculty of Medicine, University of Ljubljana, Ljubljana, Slovenia; Faculty of Tourism Studies - Turistica, University of Primorska, Portorož, Slovenia

**Keywords:** intra-bone variability, compact and cancellous bone, dual-source CT, short and sesamoid bones, bone density

## Abstract

**Background:**

Selecting the most suitable skeletal remains for genetic analysis is challenging due to the variable DNA yield across different bone types and within individual bones. Compact bone typically preserves DNA longer, whereas cancellous bones, such as those in the hands and feet, often contain higher DNA quantities. This study aimed to incorporate dual-source computed tomography (DSCT), a technique frequently utilized for assessing bone density in living subjects, into targeted DNA sampling for dry, skeletonized remains by mapping compact and cancellous regions within six small skeletal elements.

**Materials and methods:**

A total of 137 bones were analysed using an imaging protocol specifically adapted to highlight the skeletal structure of small bones. This tailored protocol involved meticulous calibration of imaging parameters. Anatomical landmarks for six distinct elements were identified, and regions of interest were selected for bone density measurement in Hounsfield units (HU).

**Results:**

Among 461 assessed regions, 312 (68%) were classified as compact bone, and 149 (32%) as cancellous bone. Given the abnormal distribution of data, statistical differences were evaluated using 95% confidence intervals, with significance indicated by non-overlapping intervals. The analysis revealed statistically significant differences between compact and cancellous bone, as well as within each type across different bones.

**Conclusions:**

DSCT proved effective in mapping the internal structure of six small skeletal elements in dry, skeletonized remains, underscoring significant intra-bone variability in density. The findings illustrate DSCT’s substantial potential for enhancing DNA sampling in forensic and paleogenetic studies, setting the stage for future research advancements.

## Introduction

Good and dependable sample selection of human skeletal remains for genetic analysis has been one of the main goals of many studies in recent years.^1–11^ Some explore the issue of bone types, their structure and varying amounts of DNA in different anatomical regions using knowledge from embryology, histology, and widely known scientific facts. In contrast, others try to explain the variations in DNA quantity by studying the effects of external factors on DNA preservation.^1,4,12^

Bones are classified into seven groups based on their shape, size, and thickness. Bone tissue is further categorised into compact and cancellous bone, which merge without clear boundaries. While the structure of long bones is relatively straightforward, the structure of other bone types, especially short and sesamoid bones, is more complex. These bones consist of thin-to-medium-thick layers of compact bone and varying amounts of cancellous bone.^13^ It was recently found that cancellous bones, such as small bones of hands and feet, contain more significant amounts of DNA than compact long bones.^4^ The petrous bone in the skull is the skeletal element with the most preserved DNA in ancient remains^14–19^ and forensic skeletal investigations.^14–16^ Selecting the right skeletal element for genetic testing is crucial, as the petrous bone may not always be available. When only short and sesamoid bones are accessible, determining which part of the bone should be used for DNA extraction becomes essential. Regardless of the bone type chosen intra-bone DNA preservation varies significantly, affecting STR typing success rates. Inter-bone DNA yield depends on bone composition, particularly the ratio of cortical to cancellous tissue, which differs across skeletal elements.^4,6–8,17,18^

To determine the best DNA sampling sites, specific examination techniques can be used, such as Attenuated Total Reflectance (ATR), Fourier Transform Infrared (FTIR) spectroscopy, and micro-computed tomography (micro-CT).^19,20^ ATR-FTIR provides detailed information about the chemical composition of bone from the surface down to a few micrometres in depth, while micro-CT offers an extremely detailed view of the microstructure of bone samples. Both techniques are insightful, however they do not allow for a comprehensive study of the bone in its entirety.

In this study, we utilised dual-source computed tomography (DSCT), a relatively new technique that can generate high-quality images of soft tissue or bone marrow by distinguishing different tissue characteristics to ensure precise and effective DNA sampling from dry, skeletonised human remains.^21–24^

Our study aimed to integrate DSCT into targeted DNA sampling to determine compact and cancellous bone regions in small skeletal elements for DNA extraction optimisation.

## Materials and methods

### Sample selection

We included 137 whole and well-preserved bones belonging to six different skeletal elements (15 patellae, 26 calcanei, 12 tali, 34 navicular bones, 29 cuboid bones, and 21 medial cuneiform bones) excavated from the same burial site, the Konfin Shaft II Mass Grave, which is described in detail in Inkret *et al*.^6^ Whole bones were subjected to CT imaging and structural analysis for the determination of compact and cancellous bone tissue for further DNA extraction optimisation.

The study was approved by The National Medical Ethics Committee of the Republic of Slovenia (0120-233/2020/3).

### CT imaging

To obtain the highest quality CT images of selected bones, a technologically high-performance DSCT device was used (Siemens Definition FORCE, Siemens, AG). The system provides a “variable focus” technology that deflects the focus with the help of a magnetic field and enables the acquisition of up to 192 reconstructed slices on a single detector. Due to the large number of channels, the detector provides a good choice between different thicknesses of the reconstructed slice from 0.4 mm to 10 mm. For the needs of the study, a new, specially adapted imaging protocol has been designed specifically to show the bone structure of small skeletal elements. Imaging in the spiral technique with high resolution was performed with the following adjusted exposure parameters: Sn150KV, mAs value to be determined from the overview image, turnaround time 1s with detector collimation 64x0.6, and 0.4 mm thickness of the reconstructed slice using the reconstruction algorithm for the bone tissue. Additionally, we used the tin filter, which optimises the photon spectrum by reducing photons with lower energies and provides a better contrast of the observed skeletal anatomy.

The raw data was analysed using an adapted protocol specific to this research, with multi-planar reconstructions of the bone sections. We used reconstructed images in the coronal plane with a thickness of 0.75 mm to obtain the desired measurements.

### Pilot study and region of interest (ROI) selection

Upon obtaining the CT images, the anatomical landmarks of each skeletal element were defined. Bone density measurements in Hounsfield units (HU) were taken for specific regions of interest (ROIs) based on the bone structure, focusing on compact and cancellous bone regions.

**FIGURE 1.**
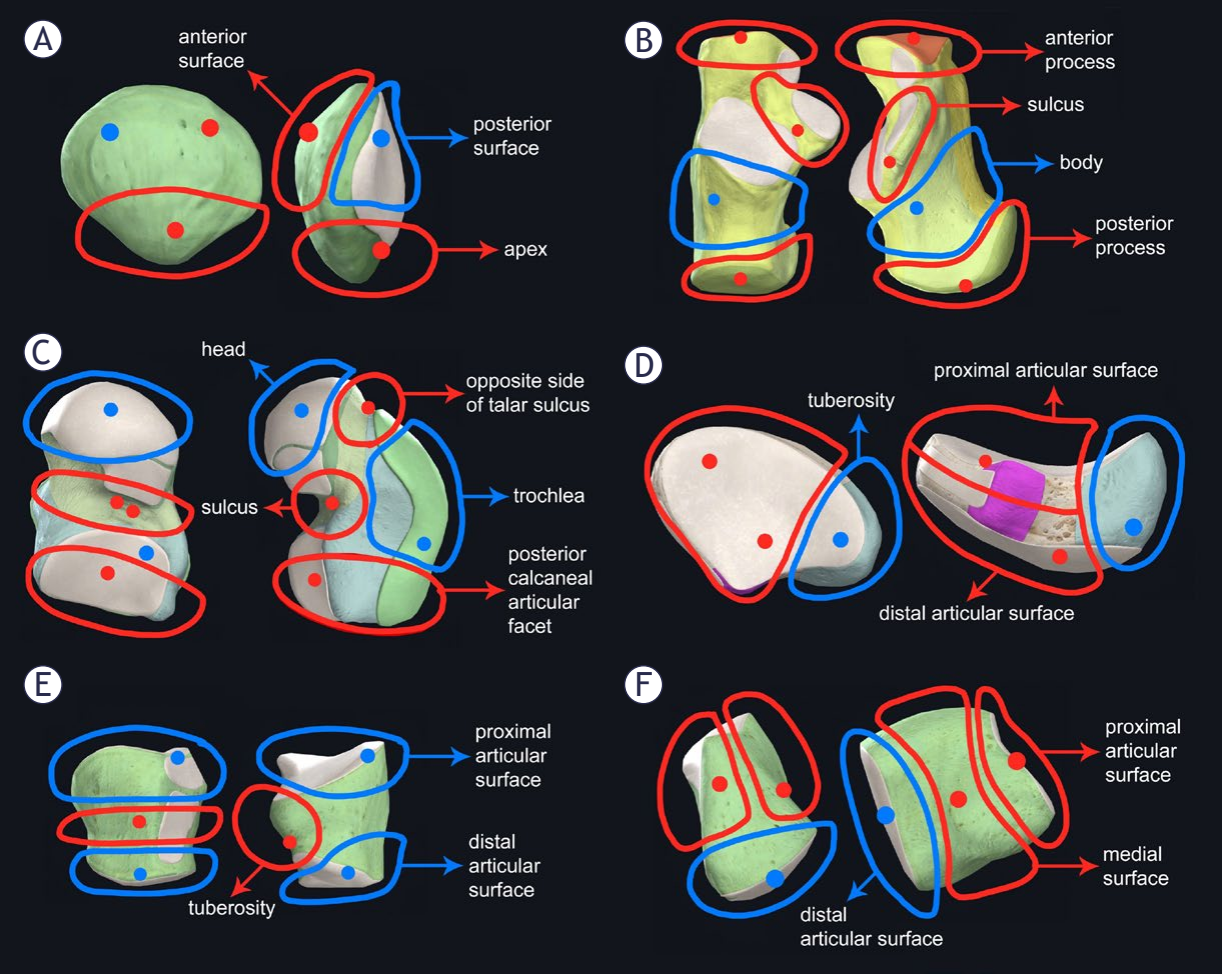
Schematic selection of regions of interest (ROIs) and parts of bones. A = patella, B = calcaneus, C = talus, D = navicular bone, E = cuboid bone, F = medial cuneiform bone

A pilot study was conducted to determine suitable HU values and ROIs for each skeletal element. In living bone tissue, HU values range from 700 for cancellous bone to 3000 for compact bone.^25^ However, because skeletonized remains lack water, the HU values were expected to be significantly lower, providing a more accurate reflection of tissue density. A study-specific HU scale for assessing the differentiation of compact and cancellous bone was established and schematic ROIs were selected (Figure 1).

Based on CT scan analysis, each skeletal element was divided into smaller parts sufficient for genetic identification sampling, in line with established DNA extraction protocols.^26^ The division of the skeletal elements into their respective parts is summarised in Table 1.

**TABLE 1.** Skeletal element parts based on CT scan analysis for genetic identification sampling

	SKELETAL ELEMENT	BONE PART
1	Patella	Apex, anterior surface, posterior surface
2	Calcaneus	Posterior process, body, sulcus, anterior process
3	Talus	Head, sulcus, opposite side of talar sulcus, trochlea, posterior calcaneal articular facet
4	Navicular bone	Proximal articular surface, distal articular surface, tuberosity
5	Cuboid bone	Proximal articular surface, tuberosity, distal articular surface
6	Medial cuneiform bone	Proximal articular surface, medial surface, distal articular surface

**FIGURE 2.**
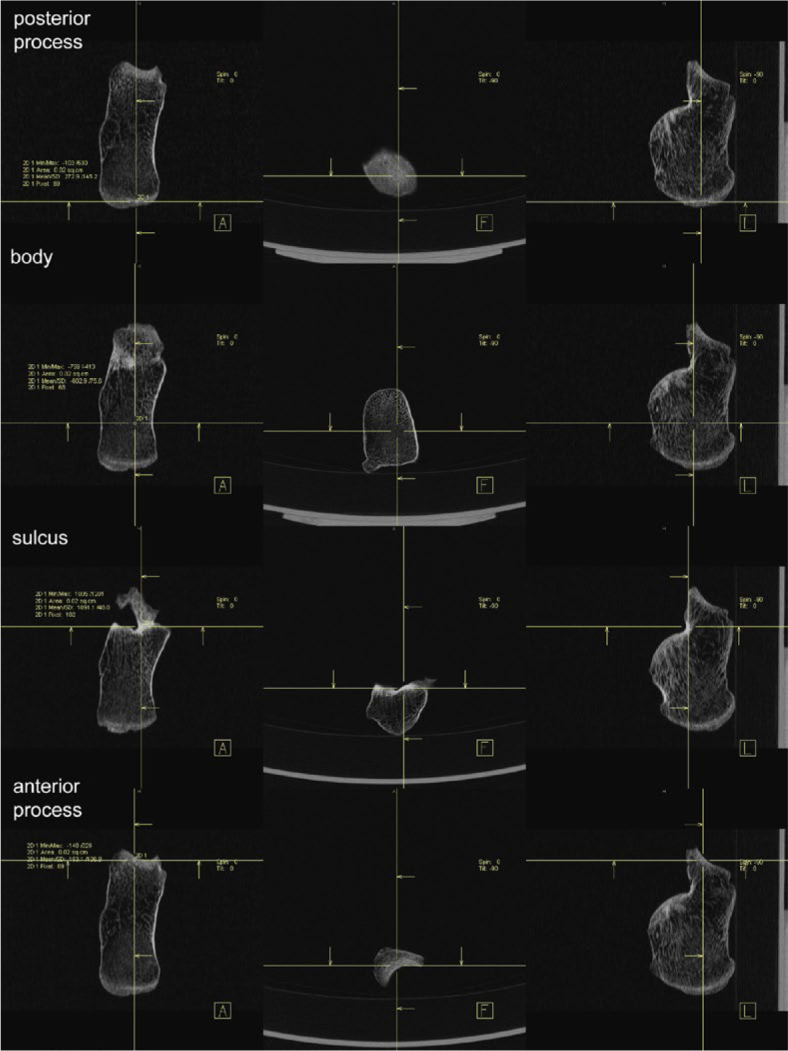
Selection of regions of interest (ROIs) on calcaneus.

A detailed view of selected ROI for all bones is shown in Supplementary material (Supplementary Figures 1–5). Calcaneus is shown as an example in Figure 2.

### Statistical analysis

All bone density values in HU from all selected ROIs were included in statistical analysis. Means and standard deviations of bone density values were calculated for each part of six skeletal elements.

To statistically assess the aim of our study, the following hypothesis was formulated: There are statistically significant differences in bone density values among selected dry bone parts of selected skeletal elements.

The normality of the measured bone density distribution was checked using the Kolmogorov-Smirnov test. Due to the abnormal distribution, statistical differences between the HU values were evaluated using 95% confidence intervals. As such, the 95% confidence intervals for means or medians^27^ were calculated using bootstrapping^28,29^ with 1,000 samples.

The differences were considered statistically significant if 95% confidence intervals did not overlap.

All statistical analyses were performed using IBM SPSS Statistics, version 26.0.

## Results

### Bone density values

The bone density values (average, minimum and maximum) in HU of 137 bones in their specific regions are presented in Table 2. The Supplementary Table 1 provides all measured bone density values.

**TABLE 2.** Bone density values (average, minimum and maximum) in Hounsfield units (HU) for individual bone parts of 6 skeletal elements

SKELETAL ELEMENT		AVERAGE HU	MIN HU VALUE	MAX HU VALUE
Patella	Apex	747.8	322.0	999.7
Patella	Anterior surface	837.9	509.4	1100.2
Patella	Posterior surface	-437.6	-592.0	-176.3
Calcaneus	Posterior process	325.6	94.5	872.5
Calcaneus	Body	-618.9	-856.4	-322.5
Calcaneus	Sulcus	900.8	299.5	1257.8
Calcaneus	Anterior process	395.4	109.0	1004.0
Talus	Head	-209.3	-464.9	-54.8
Talus	Sulcus	613.5	243.9	1012.1
Talus	Opposite side of talar sulcus	613.5	100.9	1019.2
Talus	Trochlea	-275.4	-518.0	-72.3
Talus	Posterior calcaneal articular facet	534.6	112.3	1033.2
Navicular bone	Proximal articular surface	850.6	291.2	1180.1
Navicular bone	Distal articular surface	523.0	129.2	1017.0
Navicular bone	Tuberosity	-559.9	-827.5	-163.9
Cuboid bone	Proximal articular surface	370.6	92.6	833.7
Cuboid bone	Tuberosity	506.2	103.0	860.3
Cuboid bone	Distal articular surface	-349.0	-646.7	-26.2
Medial cuneiform bone	Proximal articular surface	371.4	103.6	1140.0
Medial cuneiform bone	Medial surface	838.5	374.0	1202.5
Medial cuneiform bone	Distal articular surface	-245.8	-495.1	-107.1

The selected ROIs served as a basis for defining the compact and cancellous bone parts for each of the six skeletal elements. Out of 461 parts, 312 (68%) were compact bone and 149 (32%) cancellous bone. We identified 14 compact and seven cancellous bone parts in all six small skeletal elements.

The means, standard deviations, and tests for normality for each of the six skeletal elements are described in detail in Table 3. The highest recorded HU in the compact bone was measured in the sulcus of the calcaneus (1257.8 HU), followed by the medial surface of the medial cuneiform bone (1202.5 HU) and proximal articular surface of the navicular bone (1180.1 HU). The lowest measured HU was in the body of the calcaneus (-856.4 HU) and the tuberosity of the navicular bone (-827.5 HU) (Supplementary Table 1).

**TABLE 3.** Bone density values (mean, standard deviations, most extreme differences) and tests for normality of the distribution of Hounsfield units (HU) values for each skeletal element

SKELETAL ELEMENT		PATELLA	CALCANEUS	TALUS	NAVICULAR BONE	CUBOID BONE	MEDIAL CUNEIFORM BONE
**N**		45	104	60	102	87	63
**Normal Parameters^a^^,^^b^**	**Mean**	382.71	250.71	255.37	271.25	145.91	321.37
	**Standard deviation**	605.23	599.16	487.60	640.98	420.08	493.63
**Most extreme differences**	**Absolute**	0.28	0.15	0.14	0.17	0.12	0.14
	**Positive**	0.17	0.10	0.14	0.17	0.11	0.14
	**Negative**	-0.28	-0.15	-0.12	-0.13	-0.12	-0.09
**Test statistic**		0.28	0.15	0.14	0.17	0.12	0.14
**Asymptotic significance (2-tailed)**		< 0.01^c^	< 0.01^c^	< 0.01^c^	< 0.01^c^	< 0.01^c^	<0.01^c^

a test distribution is normal

b calculated from data

c lilliefors significance correction

### Statistical differences between bone parts

**FIGURE 3.**
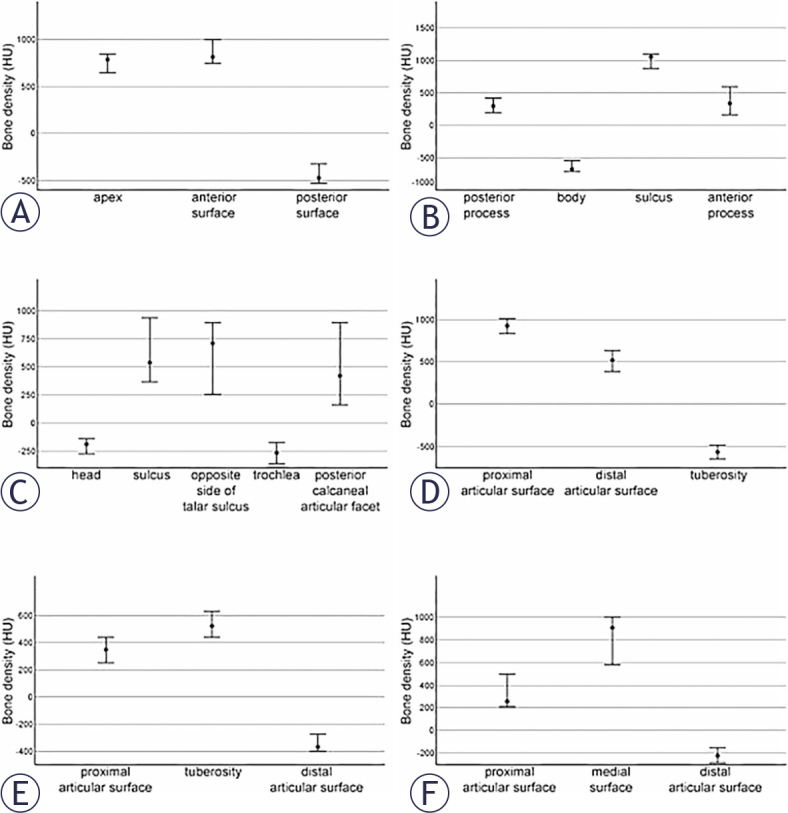
95 % confidence intervals of bone density in Hounsfield units (HU) for each skeletal element and its parts. A = patella, B = calcaneus, C = talus, D = navicular bone, E = cuboid bone, F = medial cuneiform bone

The 95% confidence intervals of measured bone density in HU for each skeletal element and its parts are presented in Figure 3. Statistical differences were found in the bone density values between compact and cancellous bone regions in all six skeletal elements (Figure 3). For the patella, more cancellous bone tissue was observed in the posterior surface, and for calcaneus in the body region. The talus’ head and trochlea are composed of more cancellous bone. In the navicular bone, tuberosity is more cancellous. The distal articular surface of the cuboid bone and medial cuneiform bone consists mainly of cancellous bone tissue.

Additionally, differences in bone density were observed within the compact bone regions of the calcaneus, navicular bone, and medial cuneiform bone (Figure 3). Specifically, we observed that bone density was higher in the calcaneal sulcus than in the other compact parts of the same bone (anterior and posterior processes).

## Discussion

Using DSCT, an already established method of measuring bone density in the general living population, we successfully mapped out the internal structural differences of dry, dead, and skeletonised human remains. Excavation from the same grave enabled comparison of bones from different skeletons, all exposed to identical decomposition and environmental conditions.

This study highlights the interesting and complex internal structure of the six small skeletal elements that we examined. CT imaging provided invaluable insight, identifying distinct bone regions that would have otherwise been overlooked. Our study focused on the smaller bones of the lower extremity. Without CT imaging, these bones may have been mistakenly divided in half, which would have mixed their compact and cancellous parts during further genetic analysis. Our results demonstrate that the internal structure of short and sesamoid bones is individual, and each skeletal element is unique. It does not always follow the belief that bones thrive under pressure, resulting in tougher and denser parts due to remodelling, microtraumas, and repeated mechanical stresses.^30–32^ Some bones had one or multiple bone islands (1 patella, 6 calcanei, 4 tali, 7 cuboid bones, 11 navicular bones and 1 medial cuneiform bone), and 3 of the anterior surfaces of patellae had prominent bony outgrowths representing proliferative enthesopathy of the quadriceps muscle.

Our study found differences in bone density among the compact parts of the calcaneus, the navicular bone, and the medial cuneiform bone. Specifically, the bone density was higher in the calcaneal sulcus than in the other compact parts of the same bone (anterior and posterior processes). The calcaneal sulcus is located in the tarsal sinus, a cylindrical cavity between the talus and calcaneus containing blood vessels, nerves, fat, and numerous ligaments crucial for ankle stability during eversion and inversion. In addition, we found that the navicular bone’s densest part is the proximal articular surface, which articulates with the talus’s head. This density may result from the physical load transfer, which results in remodelling and repairing microtraumas. According to our findings, the density of the medial cuneiform bone’s medial surface is higher than its proximal articular surface, which is likely due to the tibialis anterior muscle insertion site being on the medial surface. Our findings confirm significant intra-bone variability in bone density, even within the same type of bone structure.

This research represents a convergence of the fields of radiology and forensics. In the literature we came across, some researchers have analysed bone structure using detailed anatomy to identify individual-specific alterations or changes throughout historical periods using micro-CT and CT.^33–41^ Others have used CT to estimate age-at-death, determine sex and stature, and explore potential implications for forensics, either on forensic autopsy cases or living individuals.^42,43^ Currently, micro-CT is the most interesting and commonly used method for investigating intra-bone variability in DNA. Micro-CT allows imaging of small-sized samples (ranging from 100 nanometres to 200 millimetres) with excellent resolution, providing insights into morphology investigations. However, despite its advantages, micro-CT has certain limitations. One such disadvantage is that it is complex, demanding, and not widely available for research work. Additionally, the method does not allow for the examination of the bone in its entirety.

To the authors’ knowledge, this study is the first to analyse bone density within dry, skeletonised human remains to identify possible intra-bone variability using DSCT, an already established method of measuring bone density in the general living population. Our results provide insight into the specific internal structure of six skeletal elements as a guide for targeted DNA sampling selection.

We could only use well-preserved bones without cracks or missing areas for our material selection. As a result, some skeletal elements had fewer bones to evaluate, with only 12 talar bones and 15 patellae available. Regardless, the trend of the structure of the bones was sufficiently seen in the number of bones observed. It is possible to divide these six skeletal elements into compact and cancellous bone tissue by slicing them into thicker slices with a saw and simply observing the structure within. However, this approach could result in inconsistent slices, and valuable DNA information could be lost as parts of the bone are sawed away. Therefore, we decided not to pursue this method to avoid losing important information for subsequent further DNA analysis. While analysing CT images, we observed a simple fact: we identified more numerous but smaller, compact regions and larger cancellous regions, resulting in more compact regions per skeletal element.

## Conclusions

Our study successfully determined areas of compact and cancellous parts of 6 specific dry, dead bones using DSCT, an already established method of measuring bone density in the general living population. The bone structure is essential for determining the best sampling site for DNA extraction for molecular genetic identification in forensics and paleogenetics. Our future work could assess whether a correlation exists between CT-measured bone density and the amount of preserved DNA of small skeletal elements.

## Supplementary Material

Supplementary Material Details
