# Supplementary material for: Computed tomography differentiation of compact and cancellous bone tissue in short and sesamoid bones
Source: Radiol Oncol. 2025 Apr 11;59(3):311–8. doi: 10.2478/raon-2025-0022 (PMC12558407; doi:10.2478/raon-2025-0022)
Supplement: Supplementary file 1 — Supplementary Material Details [file raon-2025-0022_sm.pdf]

Computed tomography differentiation of compact and cancellous bone tissue in short and sesamoid bones

Ziva Miriam Gersak, Irena Zupanic-Pajnic, Eva Podovsovnik, Vladka Salapura

doi: 10.2478/raon-2025-0022

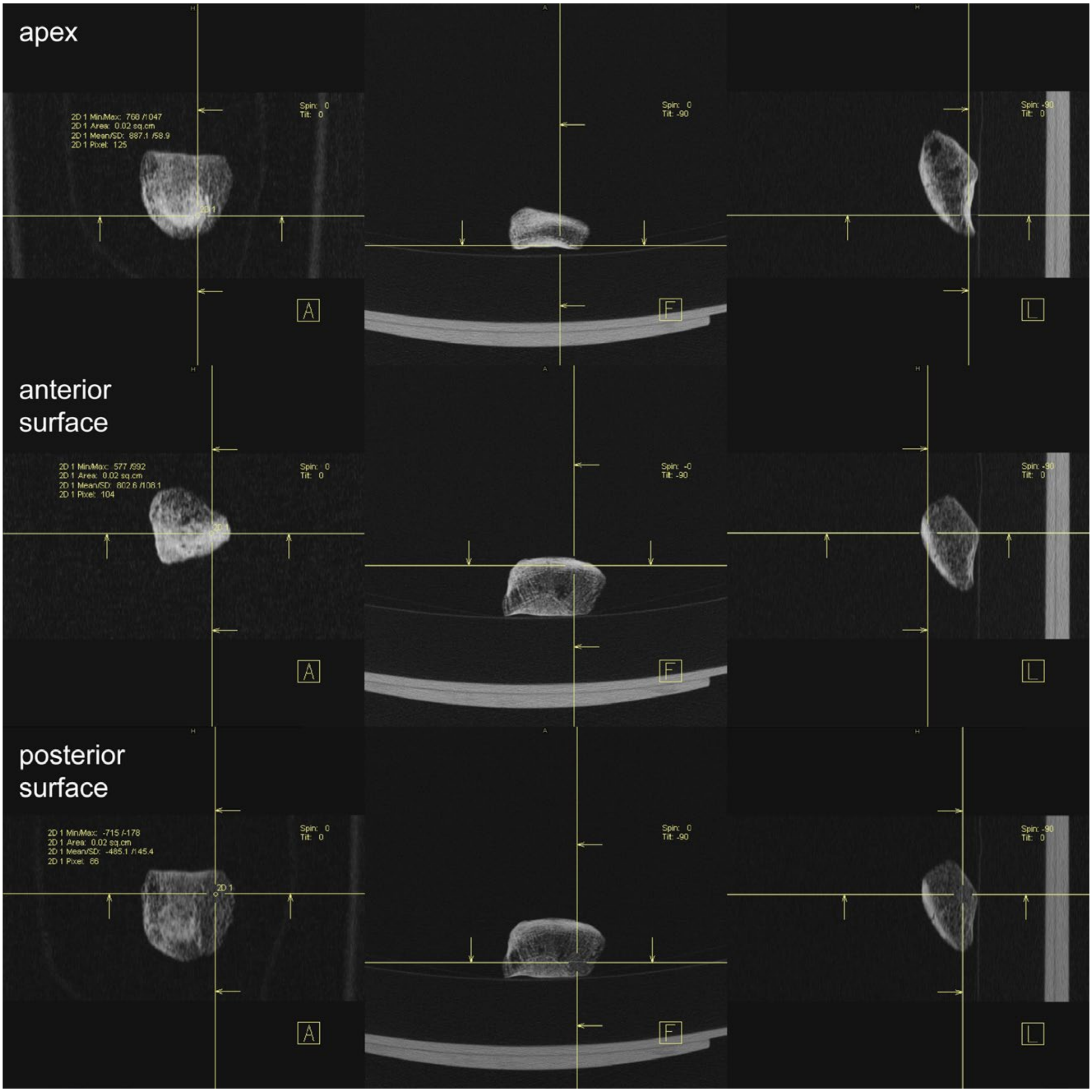

SUPPLEMENTARY FIGURE 1. Selection of regions of interest (ROIs) on patella.

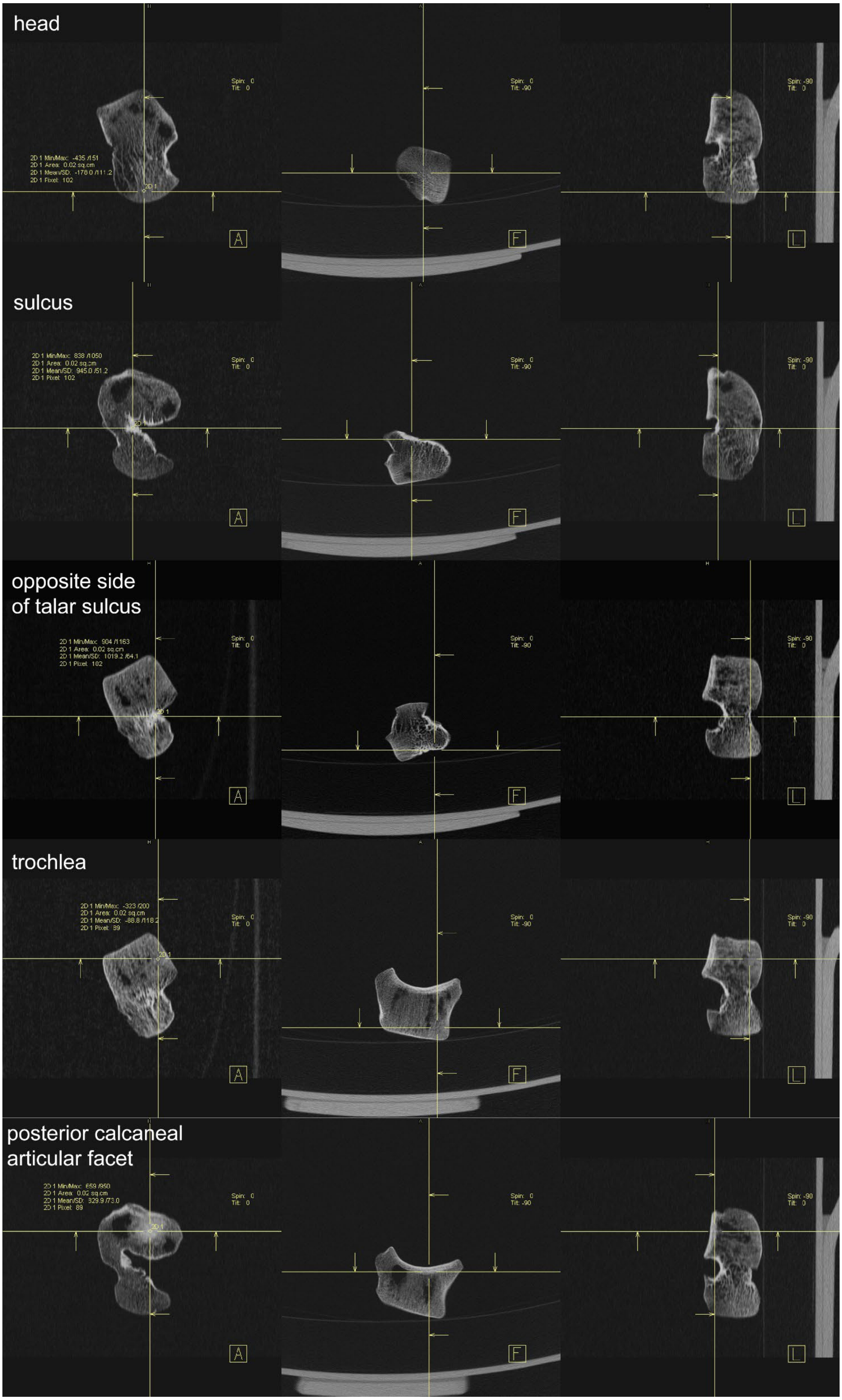

SUPPLEMENTARY FIGURE 2. Selection of regions of interest (ROIs) on talus.

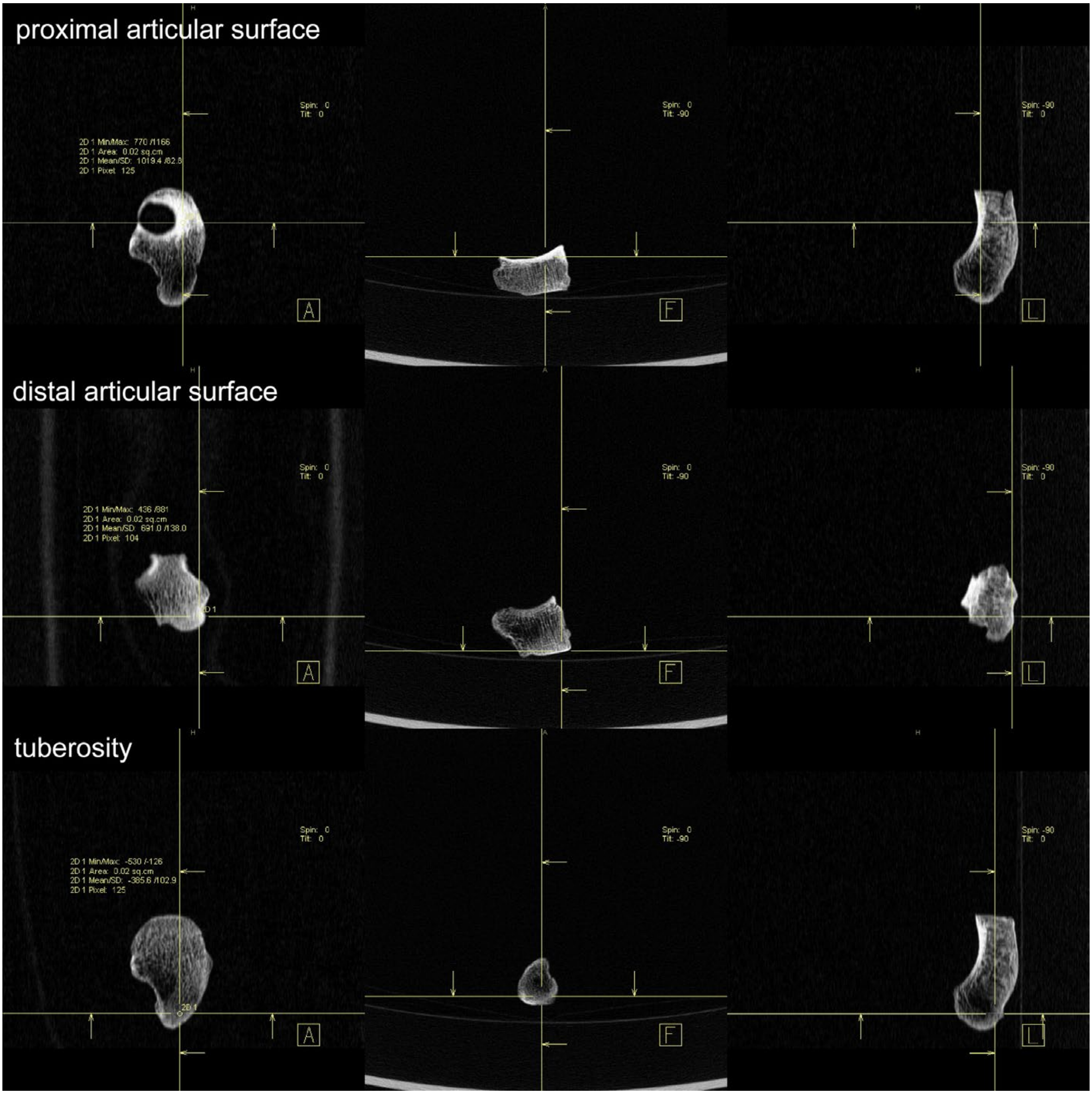

SUPPLEMENTARY FIGURE 3. Selection of regions of interest (ROIs) on the navicular bone.

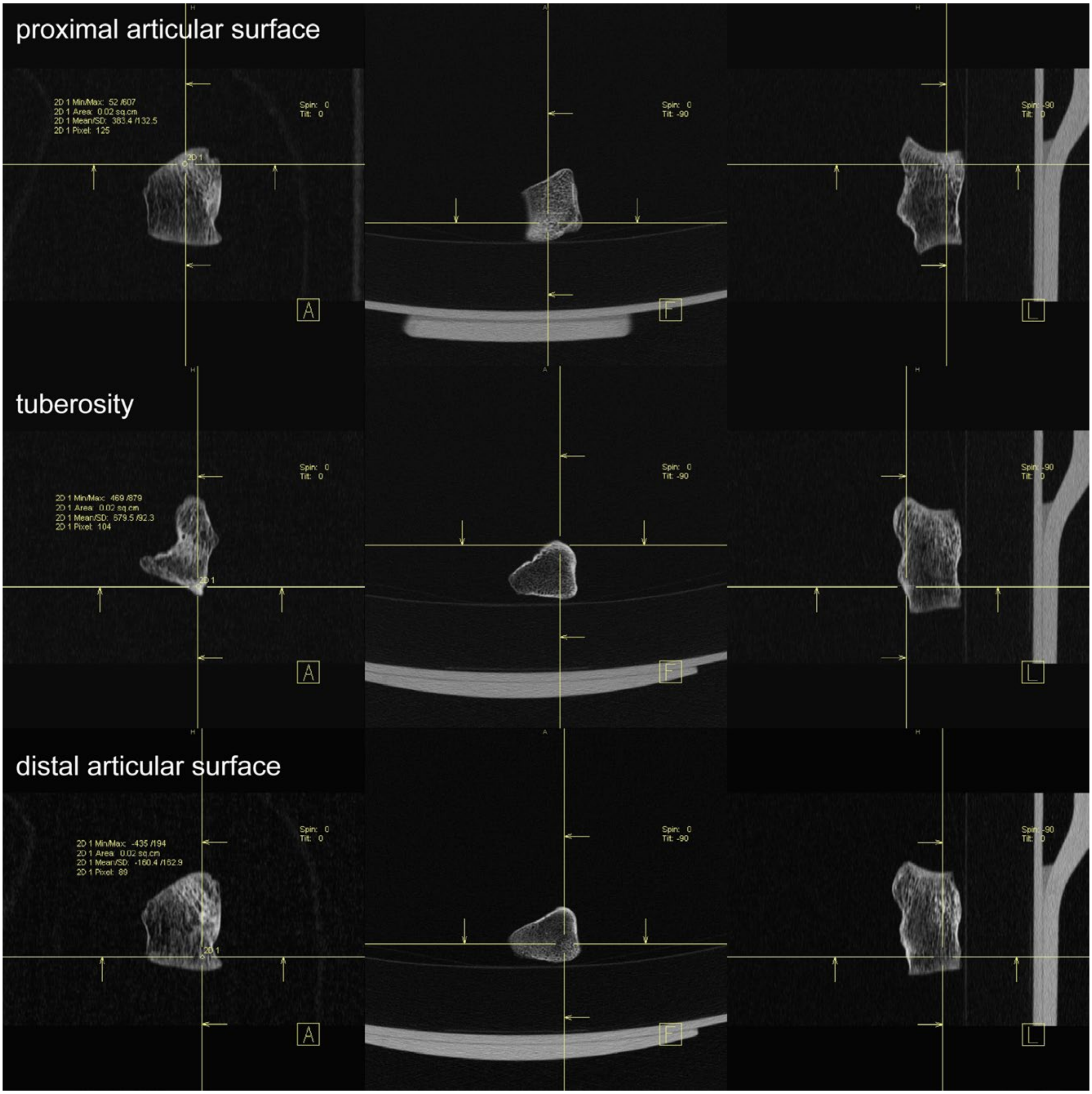

SUPPLEMENTARY FIGURE 4. Selection of regions of interest (ROIs) on the cuboid bone.

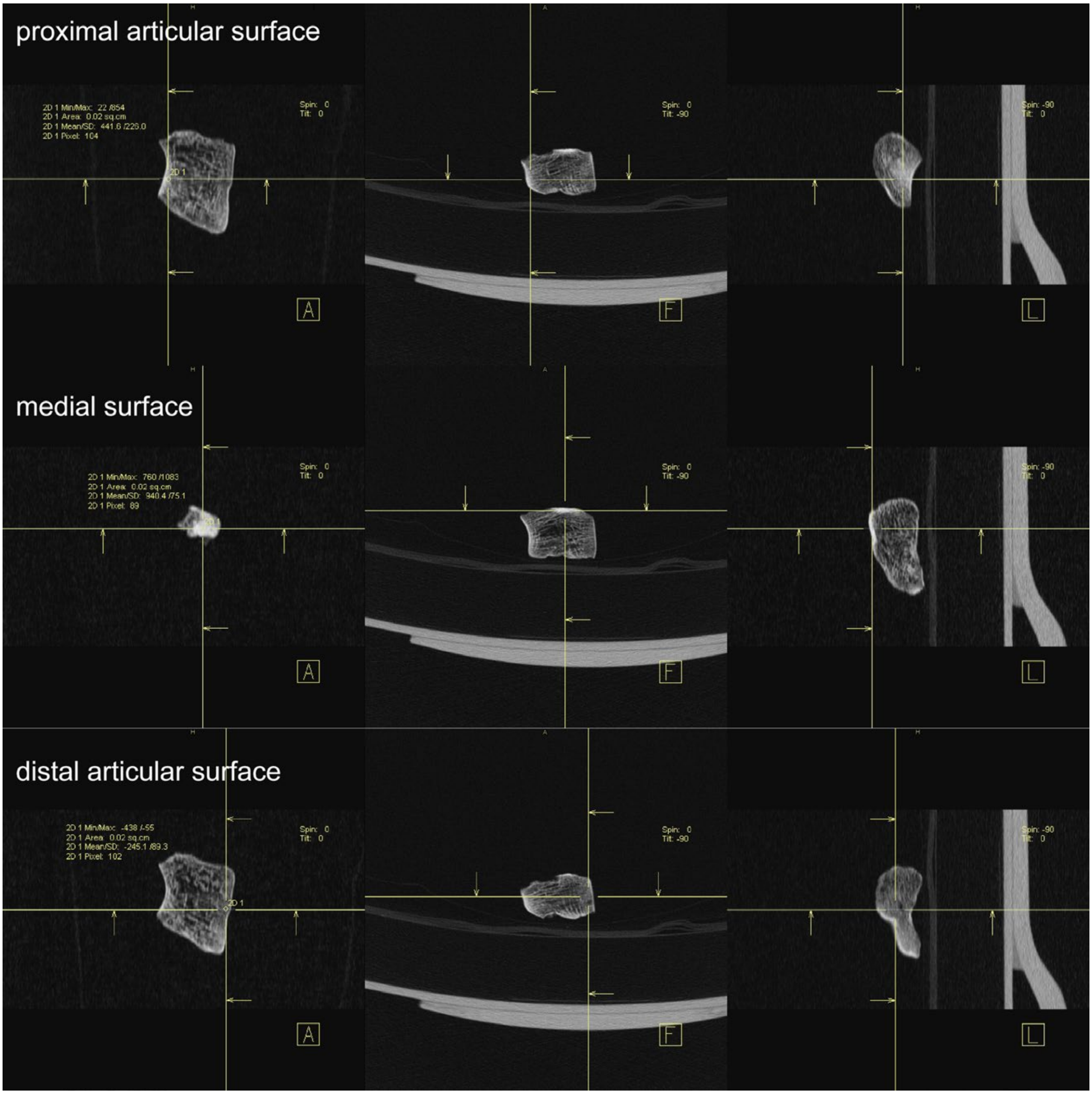

SUPPLEMENTARY FIGURE 5. Selection of regions of interest (ROIs) on the medial cuneiform bone.

SUPPLEMENTARY TABLE 1. Main database - bone density values in Hounsfield units (HU) for all individual bone parts of all 6 skeletal elements

SAMPLE	BONE	BONE SEGMENT	ROI (HU MEAN)
1	patella 1	apex	887,1
2	patella 1	anterior surface	802,6
3	patella 1	posterior surface	-485,1
4	patella 2	apex	999,7
5	patella 2	anterior surface	509,4
6	patella 2	posterior surface	-473,6
7	patella 3	apex	631,6
8	patella 3	anterior surface	747,4
9	patella 3	posterior surface	-176,3
10	patella 4	apex	801,1
11	patella 4	anterior surface	765,5
12	patella 4	posterior surface	-526,4
13	patella 5	apex	836,9
14	patella 5	anterior surface	865,2
15	patella 5	posterior surface	-592
16	patella 6	apex	647,1
17	patella 6	anterior surface	851,7
18	patella 6	posterior surface	-325,5
19	patella 7	apex	322
20	patella 7	anterior surface	644
21	patella 7	posterior surface	-400
22	patella 8	apex	928,3
23	patella 8	anterior surface	1000,1
24	patella 8	posterior surface	-490,9
25	patella 9	apex	707,8
26	patella 9	anterior surface	1003,7
27	patella 9	posterior surface	-576,5
28	patella 10	apex	703,7
29	patella 10	anterior surface	750,8
30	patella 10	posterior surface	-528
31	patella 11	apex	805,1
32	patella 11	anterior surface	1005,7
33	patella 11	posterior surface	-317,7
34	patella 12	apex	786,5
35	patella 12	anterior surface	1100,2
36	patella 12	posterior surface	-226,2
37	patella 13	apex	629,7
38	patella 13	anterior surface	907,1
39	patella 13	posterior surface	-422,8
40	patella 14	apex	827,3
41	patella 14	anterior surface	810,3
42	patella 14	posterior surface	-445,2
43	patella 15	apex	703,5
44	patella 15	anterior surface	805,3
45	patella 15	posterior surface	-578,4
46	calcaneus 1	posterior process	317,1
47	calcaneus 1	body	-465,1
48	calcaneus 1	sulcus	360,6
49	calcaneus 1	anterior process	256,3
50	calcaneus 2	posterior process	425,3
51	calcaneus 2	body	-668,8
52	calcaneus 2	sulcus	299,5
53	calcaneus 2	anterior process	135,5
54	calcaneus 3	posterior process	198,2
55	calcaneus 3	body	-455,6
56	calcaneus 3	sulcus	1049,3
57	calcaneus 3	anterior process	800,7
58	calcaneus 4	posterior process	272,9
59	calcaneus 4	body	-602,9

60	calcaneus 4	sulcus	1091,1
61	calcaneus 4	anterior process	153,1
62	calcaneus 5	posterior process	872,5
63	calcaneus 5	body	-365,2
64	calcaneus 5	sulcus	1121,3
65	calcaneus 5	anterior process	334,9
66	calcaneus 6	posterior process	108,4
67	calcaneus 6	body	-322,5
68	calcaneus 6	sulcus	1074,1
69	calcaneus 6	anterior process	649,2
70	calcaneus 7	posterior process	94,5
71	calcaneus 7	body	-709,5
72	calcaneus 7	sulcus	594,2
73	calcaneus 7	anterior process	286,2
74	calcaneus 8	posterior process	165,3
75	calcaneus 8	body	-426,5
76	calcaneus 8	sulcus	875,2
77	calcaneus 8	anterior process	454,8
78	calcaneus 9	posterior process	361,2
79	calcaneus 9	body	-456,7
80	calcaneus 9	sulcus	1149,7
81	calcaneus 9	anterior process	655
82	calcaneus 10	posterior process	418,5
83	calcaneus 10	body	-401,7
84	calcaneus 10	sulcus	1072
85	calcaneus 10	anterior process	678,3
86	calcaneus 11	posterior process	139,4
87	calcaneus 11	body	-621,1
88	calcaneus 11	sulcus	1137,3
89	calcaneus 11	anterior process	115,9
90	calcaneus 12	posterior process	107,2
91	calcaneus 12	body	-687
92	calcaneus 12	sulcus	1257,8
93	calcaneus 12	anterior process	354,5
94	calcaneus 13	posterior process	185,2
95	calcaneus 13	body	-798,4
96	calcaneus 13	sulcus	437,1
97	calcaneus 13	anterior process	109
98	calcaneus 14	posterior process	218,8
99	calcaneus 14	body	-699,6
100	calcaneus 14	sulcus	544,6
101	calcaneus 14	anterior process	132,4
102	calcaneus 15	posterior process	417,9
103	calcaneus 15	body	-654,8
104	calcaneus 15	sulcus	1021,1
105	calcaneus 15	anterior process	218,4
106	calcaneus 16	posterior process	293,1
107	calcaneus 16	body	-729
108	calcaneus 16	sulcus	946,8
109	calcaneus 16	anterior process	144,9
110	calcaneus 17	posterior process	170
111	calcaneus 17	body	-856,4
112	calcaneus 17	sulcus	468,2
113	calcaneus 17	anterior process	156,7
114	calcaneus 18	posterior process	728,6
115	calcaneus 18	body	-730,7
116	calcaneus 18	sulcus	1003,4
117	calcaneus 18	anterior process	200,7
118	calcaneus 19	posterior process	569,1
119	calcaneus 19	body	-739,5

120	calcaneus 19	sulcus	1061,8
121	calcaneus 19	anterior process	688,9
122	calcaneus 20	posterior process	119,7
123	calcaneus 20	body	-670,6
124	calcaneus 20	sulcus	1121,1
125	calcaneus 20	anterior process	408,6
126	calcaneus 21	posterior process	418,6
127	calcaneus 21	body	-716,1
128	calcaneus 21	sulcus	308,3
129	calcaneus 21	anterior process	109,4
130	calcaneus 22	posterior process	359,3
131	calcaneus 22	body	-532,1
132	calcaneus 22	sulcus	1048
133	calcaneus 22	anterior process	942,6
134	calcaneus 23	posterior process	238,8
135	calcaneus 23	body	-716,1
136	calcaneus 23	sulcus	1099,8
137	calcaneus 23	anterior process	1004
138	calcaneus 24	posterior process	580,9
139	calcaneus 24	body	-598,5
140	calcaneus 24	sulcus	1128,3
141	calcaneus 24	anterior process	589,3
142	calcaneus 25	posterior process	304,8
143	calcaneus 25	body	-705,5
144	calcaneus 25	sulcus	1061,5
145	calcaneus 25	anterior process	373
146	calcaneus 26	posterior process	380,1
147	calcaneus 26	body	-760,6
148	calcaneus 26	sulcus	1087,6
149	calcaneus 26	anterior process	327,2
150	talus 1	head	-157,2
151	talus 1	sulcus	374,9
152	talus 1	opposite side of talar sulcus	361,8
153	talus 1	throchlea	-261,3
154	talus 1	posterior cancaneal articular facet	168,1
155	talus 2	head	-115,3
156	talus 2	sulcus	609,5
157	talus 2	opposite side of talar sulcus	812
158	talus 2	throchlea	-264,2
159	talus 2	posterior cancaneal articular facet	1033,2
160	talus 3	head	-464,9
161	talus 3	sulcus	975,5
162	talus 3	opposite side of talar sulcus	902,4
163	talus 3	throchlea	-177,2
164	talus 3	posterior cancaneal articular facet	872
165	talus 4	head	-178
166	talus 4	sulcus	945
167	talus 4	opposite side of talar sulcus	1019,2
168	talus 4	throchlea	-88,8
169	talus 4	posterior cancaneal articular facet	829,9
170	talus 5	head	-254,6
171	talus 5	sulcus	761,6
172	talus 5	opposite side of talar sulcus	1018,7
173	talus 5	throchlea	-72,3
174	talus 5	posterior cancaneal articular facet	1024,7
175	talus 6	head	-304,5
176	talus 6	sulcus	243,9
177	talus 6	opposite side of talar sulcus	262,2
178	talus 6	throchlea	-420,3
179	talus 6	posterior cancaneal articular facet	318,3

180	talus 7	head	-142
181	talus 7	sulcus	329,4
182	talus 7	opposite side of talar sulcus	187,8
183	talus 7	throchlea	-365,6
184	talus 7	posterior cancaneal articular facet	112,3
185	talus 8	head	-192
186	talus 8	sulcus	464,8
187	talus 8	opposite side of talar sulcus	100,9
188	talus 8	throchlea	-273,9
189	talus 8	posterior cancaneal articular facet	244
190	talus 9	head	-278,3
191	talus 9	sulcus	865,8
192	talus 9	opposite side of talar sulcus	905,2
193	talus 9	throchlea	-286
194	talus 9	posterior cancaneal articular facet	528,7
195	talus 10	head	-54,8
196	talus 10	sulcus	367
197	talus 10	opposite side of talar sulcus	618,6
198	talus 10	throchlea	-518
199	talus 10	posterior cancaneal articular facet	156,3
200	talus 11	head	-144,8
201	talus 11	sulcus	412
202	talus 11	opposite side of talar sulcus	288,9
203	talus 11	throchlea	-246,9
204	talus 11	posterior cancaneal articular facet	224,9
205	talus 12	head	-225,8
206	talus 12	sulcus	1012,1
207	talus 12	opposite side of talar sulcus	884
208	talus 12	throchlea	-329,8
209	talus 12	posterior cancaneal articular facet	903,1
210	navicular bone 1	tuberosity	-163,9
211	navicular bone 1	proximal articular surface	777,9
212	navicular bone 1	distal articular surface	421
213	navicular bone 2	tuberosity	-385,6
214	navicular bone 2	proximal articular surface	1019,4
215	navicular bone 2	distal articular surface	691
216	navicular bone 3	tuberosity	-624,2
217	navicular bone 3	proximal articular surface	697
218	navicular bone 3	distal articular surface	896,2
219	navicular bone 4	tuberosity	-827,5
220	navicular bone 4	proximal articular surface	755,8
221	navicular bone 4	distal articular surface	419,2
222	navicular bone 5	tuberosity	-595,4
223	navicular bone 5	proximal articular surface	846,2
224	navicular bone 5	distal articular surface	1017
225	navicular bone 6	tuberosity	-716,1
226	navicular bone 6	proximal articular surface	741,8
227	navicular bone 6	distal articular surface	512,2
228	navicular bone 7	tuberosity	-662,8
229	navicular bone 7	proximal articular surface	1180,1
230	navicular bone 7	distal articular surface	593,5
231	navicular bone 8	tuberosity	-553,4
232	navicular bone 8	proximal articular surface	1087,5
233	navicular bone 8	distal articular surface	530,4
234	navicular bone 9	tuberosity	-568,3
235	navicular bone 9	proximal articular surface	929,5
236	navicular bone 9	distal articular surface	736,8
237	navicular bone 10	tuberosity	-502,8
238	navicular bone 10	proximal articular surface	1060,9
239	navicular bone 10	distal articular surface	209,3

240	navicular bone 11	tuberosity	-565,4
241	navicular bone 11	proximal articular surface	1010,2
242	navicular bone 11	distal articular surface	334,6
243	navicular bone 12	tuberosity	-653,7
244	navicular bone 12	proximal articular surface	873,1
245	navicular bone 12	distal articular surface	219,4
246	navicular bone 13	tuberosity	-559,5
247	navicular bone 13	proximal articular surface	1010,4
248	navicular bone 13	distal articular surface	541,1
249	navicular bone 14	tuberosity	-730,4
250	navicular bone 14	proximal articular surface	1015,8
251	navicular bone 14	distal articular surface	521,4
252	navicular bone 15	tuberosity	-477,6
253	navicular bone 15	proximal articular surface	954
254	navicular bone 15	distal articular surface	327
255	navicular bone 16	tuberosity	-594
256	navicular bone 16	proximal articular surface	837,6
257	navicular bone 16	distal articular surface	384,2
258	navicular bone 17	tuberosity	-384,3
259	navicular bone 17	proximal articular surface	1046,3
260	navicular bone 17	distal articular surface	866,4
261	navicular bone 18	tuberosity	-642,8
262	navicular bone 18	proximal articular surface	842,6
263	navicular bone 18	distal articular surface	150,2
264	navicular bone 19	tuberosity	-685,5
265	navicular bone 19	proximal articular surface	771,9
266	navicular bone 19	distal articular surface	604,2
267	navicular bone 20	tuberosity	-387,9
268	navicular bone 20	proximal articular surface	1044,5
269	navicular bone 20	distal articular surface	943,8
270	navicular bone 21	tuberosity	-431,8
271	navicular bone 21	proximal articular surface	957,3
272	navicular bone 21	distal articular surface	651,8
273	navicular bone 22	tuberosity	-564,3
274	navicular bone 22	proximal articular surface	918
275	navicular bone 22	distal articular surface	525,9
276	navicular bone 23	tuberosity	-540,5
277	navicular bone 23	proximal articular surface	969,2
278	navicular bone 23	distal articular surface	432,3
279	navicular bone 24	tuberosity	-432,2
280	navicular bone 24	proximal articular surface	1005,6
281	navicular bone 24	distal articular surface	847,2
282	navicular bone 25	tuberosity	-433,1
283	navicular bone 25	proximal articular surface	921,8
284	navicular bone 25	distal articular surface	626,1
285	navicular bone 26	tuberosity	-392,8
286	navicular bone 26	proximal articular surface	1017,6
287	navicular bone 26	distal articular surface	823,8
288	navicular bone 27	tuberosity	-660,8
289	navicular bone 27	proximal articular surface	1018
290	navicular bone 27	distal articular surface	630,2
291	navicular bone 28	tuberosity	-743,7
292	navicular bone 28	proximal articular surface	891,6
293	navicular bone 28	distal articular surface	298,4
294	navicular bone 29	tuberosity	-470,8
295	navicular bone 29	proximal articular surface	629,2
296	navicular bone 29	distal articular surface	396,8
297	navicular bone 30	tuberosity	-573,1
298	navicular bone 30	proximal articular surface	832,3
299	navicular bone 30	distal articular surface	271,4

300	navicular bone 31	tuberosity	-749,2
301	navicular bone 31	proximal articular surface	294,3
302	navicular bone 31	distal articular surface	204,5
303	navicular bone 32	tuberosity	-464,3
304	navicular bone 32	proximal articular surface	369
305	navicular bone 32	distal articular surface	129,2
306	navicular bone 33	tuberosity	-650,9
307	navicular bone 33	proximal articular surface	302,5
308	navicular bone 33	distal articular surface	197,4
309	navicular bone 34	tuberosity	-646,9
310	navicular bone 34	proximal articular surface	291,2
311	navicular bone 34	distal articular surface	828,9
312	cuboid bone 1	proximal articular surface	383,4
313	cuboid bone 1	tuberosity	679,5
314	cuboid bone 1	distal articular surface	-160,4
315	cuboid bone 2	proximal articular surface	349,4
316	cuboid bone 2	tuberosity	467,8
317	cuboid bone 2	distal articular surface	-501,2
318	cuboid bone 3	proximal articular surface	211,9
319	cuboid bone 3	tuberosity	468,9
320	cuboid bone 3	distal articular surface	-555,5
321	cuboid bone 4	proximal articular surface	249,3
322	cuboid bone 4	tuberosity	304,5
323	cuboid bone 4	distal articular surface	-219,4
324	cuboid bone 5	proximal articular surface	300,5
325	cuboid bone 5	tuberosity	605,6
326	cuboid bone 5	distal articular surface	-267,3
327	cuboid bone 6	proximal articular surface	620,3
328	cuboid bone 6	tuberosity	605,5
329	cuboid bone 6	distal articular surface	-646,7
330	cuboid bone 7	proximal articular surface	435,5
331	cuboid bone 7	tuberosity	450,4
332	cuboid bone 7	distal articular surface	-489,8
333	cuboid bone 8	proximal articular surface	258,4
334	cuboid bone 8	tuberosity	618,7
335	cuboid bone 8	distal articular surface	-163,3
336	cuboid bone 9	proximal articular surface	170,2
337	cuboid bone 9	tuberosity	434,4
338	cuboid bone 9	distal articular surface	-313,4
339	cuboid bone 10	proximal articular surface	806,5
340	cuboid bone 10	tuberosity	860,3
341	cuboid bone 10	distal articular surface	-534,3
342	cuboid bone 11	proximal articular surface	103,8
343	cuboid bone 11	tuberosity	520,6
344	cuboid bone 11	distal articular surface	-403,2
345	cuboid bone 12	proximal articular surface	454,5
346	cuboid bone 12	tuberosity	651,4
347	cuboid bone 12	distal articular surface	-53,2
348	cuboid bone 13	proximal articular surface	447,4
349	cuboid bone 13	tuberosity	632,5
350	cuboid bone 13	distal articular surface	-352,1
351	cuboid bone 14	proximal articular surface	206,6
352	cuboid bone 14	tuberosity	298,4
353	cuboid bone 14	distal articular surface	-391,1
354	cuboid bone 15	proximal articular surface	773,8
355	cuboid bone 15	tuberosity	632,5
356	cuboid bone 15	distal articular surface	-271,5
357	cuboid bone 16	proximal articular surface	92,6
358	cuboid bone 16	tuberosity	659,5
359	cuboid bone 16	distal articular surface	-540,5

360	cuboid bone 17	proximal articular surface	284
361	cuboid bone 17	tuberosity	736,4
362	cuboid bone 17	distal articular surface	-337,3
363	cuboid bone 18	proximal articular surface	375,7
364	cuboid bone 18	tuberosity	504,4
365	cuboid bone 18	distal articular surface	-393,6
366	cuboid bone 19	proximal articular surface	352,1
367	cuboid bone 19	tuberosity	788,8
368	cuboid bone 19	distal articular surface	-369,4
369	cuboid bone 20	proximal articular surface	220,7
370	cuboid bone 20	tuberosity	526
371	cuboid bone 20	distal articular surface	-420,7
372	cuboid bone 21	proximal articular surface	410,9
373	cuboid bone 21	tuberosity	744,2
374	cuboid bone 21	distal articular surface	-254,1
375	cuboid bone 22	proximal articular surface	202,3
376	cuboid bone 22	tuberosity	105,4
377	cuboid bone 22	distal articular surface	-219,8
378	cuboid bone 23	proximal articular surface	326,3
379	cuboid bone 23	tuberosity	258,7
380	cuboid bone 23	distal articular surface	-296,2
381	cuboid bone 24	proximal articular surface	513,6
382	cuboid bone 24	tuberosity	278,5
383	cuboid bone 24	distal articular surface	-426,8
384	cuboid bone 25	proximal articular surface	106,4
385	cuboid bone 25	tuberosity	246,5
386	cuboid bone 25	distal articular surface	-377
387	cuboid bone 26	proximal articular surface	833,7
388	cuboid bone 26	tuberosity	493,9
389	cuboid bone 26	distal articular surface	-396,5
390	cuboid bone 27	proximal articular surface	346
391	cuboid bone 27	tuberosity	103
392	cuboid bone 27	distal articular surface	-360,3
393	cuboid bone 28	proximal articular surface	299,4
394	cuboid bone 28	tuberosity	233,5
395	cuboid bone 28	distal articular surface	-381,4
396	cuboid bone 29	proximal articular surface	612,9
397	cuboid bone 29	tuberosity	768,8
398	cuboid bone 29	distal articular surface	-26,2
399	medial cuneiform bone 1	proximal articular surface	103,6
400	medial cuneiform bone 1	medial surface	861
401	medial cuneiform bone 1	distal articular surface	-236,1
402	medial cuneiform bone 2	proximal articular surface	121
403	medial cuneiform bone 2	medial surface	896,5
404	medial cuneiform bone 2	distal articular surface	-139,1
405	medial cuneiform bone 3	proximal articular surface	441,6
406	medial cuneiform bone 3	medial surface	940,4
407	medial cuneiform bone 3	distal articular surface	-245,1
408	medial cuneiform bone 4	proximal articular surface	301
409	medial cuneiform bone 4	medial surface	576,5
410	medial cuneiform bone 4	distal articular surface	-146
411	medial cuneiform bone 5	proximal articular surface	292,6
412	medial cuneiform bone 5	medial surface	583,4
413	medial cuneiform bone 5	distal articular surface	-495,1
414	medial cuneiform bone 6	proximal articular surface	237,2
415	medial cuneiform bone 6	medial surface	1037,5
416	medial cuneiform bone 6	distal articular surface	-290,8
417	medial cuneiform bone 7	proximal articular surface	484,1
418	medial cuneiform bone 7	medial surface	374
419	medial cuneiform bone 7	distal articular surface	-145,2

420	medial cuneiform bone 8	proximal articular surface	261,3
421	medial cuneiform bone 8	medial surface	935,3
422	medial cuneiform bone 8	distal articular surface	-426,4
423	medial cuneiform bone 9	proximal articular surface	211,5
424	medial cuneiform bone 9	medial surface	569,2
425	medial cuneiform bone 9	distal articular surface	-205,4
426	medial cuneiform bone 10	proximal articular surface	256,6
427	medial cuneiform bone 10	medial surface	1002,5
428	medial cuneiform bone 10	distal articular surface	-231
429	medial cuneiform bone 11	proximal articular surface	1140
430	medial cuneiform bone 11	medial surface	1090,1
431	medial cuneiform bone 11	distal articular surface	-228,8
432	medial cuneiform bone 12	proximal articular surface	665,8
433	medial cuneiform bone 12	medial surface	816,2
434	medial cuneiform bone 12	distal articular surface	-222
435	medial cuneiform bone 13	proximal articular surface	217,6
436	medial cuneiform bone 13	medial surface	993,4
437	medial cuneiform bone 13	distal articular surface	-476,3
438	medial cuneiform bone 14	proximal articular surface	497,3
439	medial cuneiform bone 14	medial surface	530,4
440	medial cuneiform bone 14	distal articular surface	-156,1
441	medial cuneiform bone 15	proximal articular surface	239,2
442	medial cuneiform bone 15	medial surface	950,1
443	medial cuneiform bone 15	distal articular surface	-176,2
444	medial cuneiform bone 16	proximal articular surface	198,8
445	medial cuneiform bone 16	medial surface	685,3
446	medial cuneiform bone 16	distal articular surface	-407
447	medial cuneiform bone 17	proximal articular surface	200,1
448	medial cuneiform bone 17	medial surface	910,8
449	medial cuneiform bone 17	distal articular surface	-213,4
450	medial cuneiform bone 18	proximal articular surface	552,9
451	medial cuneiform bone 18	medial surface	1118,9
452	medial cuneiform bone 18	distal articular surface	-281,2
453	medial cuneiform bone 19	proximal articular surface	542
454	medial cuneiform bone 19	medial surface	1064,2
455	medial cuneiform bone 19	distal articular surface	-126,6
456	medial cuneiform bone 20	proximal articular surface	731,4
457	medial cuneiform bone 20	medial surface	1202,5
458	medial cuneiform bone 20	distal articular surface	-107,1
459	medial cuneiform bone 21	proximal articular surface	104
460	medial cuneiform bone 21	medial surface	470,9
461	medial cuneiform bone 21	distal articular surface	-207,6
